# Antioxidants in Sunscreens: Which and What For?

**DOI:** 10.3390/antiox12010138

**Published:** 2023-01-06

**Authors:** Ana Jesus, Sandra Mota, Ana Torres, Maria T. Cruz, Emília Sousa, Isabel F. Almeida, Honorina Cidade

**Affiliations:** 1 Associate Laboratory i4HB—Institute for Health and Bioeconomy, Faculty of Pharmacy, University of Porto, 4050-313 Porto, Portugal; 2 UCIBIO—Applied Molecular Biosciences Unit, MedTech, Laboratory of Pharmaceutical Technology, Department of Drug Sciences, Faculty of Pharmacy, University of Porto, 4050-313 Porto, Portugal; 3 Faculty of Pharmacy, University of Coimbra, 3004-531 Coimbra, Portugal; 4 Center for Neuroscience and Cell Biology, 3004-504 Coimbra, Portugal; 5 Laboratory of Organic and Pharmaceutical Chemistry, Department of Chemical Sciences, Faculty of Pharmacy, University of Porto, 4050-313 Porto, Portugal; 6 CIIMAR—Interdisciplinary Center of Marine and Environmental Research, Avenida General Norton de Matos, S/N, 4450-208 Matosinhos, Portugal

**Keywords:** sunscreens, antioxidants, trends, scientific evidence, photoprotection

## Abstract

Ultraviolet (UV) radiation promotes the generation of reactive oxygen species (ROS) and nitrogen species (RNS), resulting in skin damage. Cosmetic industries have adopted a strategy to incorporate antioxidants in sunscreen formulations to prevent or minimize UV-induced oxidative damage, boost photoprotection effectiveness, and mitigate skin photoaging. Many antioxidants are naturally derived, mainly from terrestrial plants; however, marine organisms have been increasingly explored as a source of new potent antioxidant molecules. This work aims to characterize the frequency of the use of antioxidants in commercial sunscreens. Photoprotective formulations currently marketed in parapharmacies and pharmacies were analyzed with respect to the composition described on the label. As a result, pure compounds with antioxidant activity were found. The majority of sunscreen formulations contained antioxidants, with vitamin E and its derivatives the most frequent. A more thorough analysis of these antioxidants is also provided, unveiling the top antioxidant ingredients found in sunscreens. A critical appraisal of the scientific evidence regarding their effectiveness is also performed. In conclusion, this work provides an up-to-date overview of the use of antioxidants in commercial sunscreens for a better understanding of the advantages associated with their use in photoprotective formulations.

## 1. Introduction

Excessive exposure to ultraviolet radiation (UVR) is associated with serious health risks such as UV-induced skin damage, skin photoaging (atrophy, pigmentary changes, and wrinkles), solar sunburn, skin sensitization, and malignancy [1]. UV skin damage depends on the duration and intensity of UVR exposure, particularly UVA (320–400 nm), UVB (290–320 nm), visible light, and infrared (IR) radiation [2]. UVB radiation only has the capacity to reach the epidermis and is responsible for the largest number of deleterious occurrences on the skin [3,4]. However, UVA radiation is the main promoter of skin photodamage, penetrating the dermis and contributing to the production and release of reactive oxygen (ROS) and nitrogen (RNS) species [4,5]. Skin oxidative stress causes cellular damage, as well as the activation of matrix metalloproteinases (MMPs), which break down matrix proteins, including collagen and elastin, resulting in the reduction in skin hydration and elasticity and acceleration of wrinkle formation [6,7].

Several studies have supported the benefit of the daily use of sunscreens to prevent solar skin damage [8,9,10,11]. Most sunscreens are composed of a combination of UV filters, to ensure broad spectrum protection and improve the solar protection factor (SPF) value. Regarding their capacity to absorb UVR, UV filters can be categorized as UVA, UVB, or broad-spectrum UV filters (UVA and UVB). Additionally, UV filters can be distinguished as organic, which can only absorb UVR, or inorganic, which can also reflect and scatter it [12]. Furthermore, sunscreens usually incorporate antioxidant substances to stabilize the formulation and confer additional protection against UV-mediated oxidative stress [3].

According to their mechanisms of action, antioxidants are generally classified as primary, secondary, or multifunctional [13]. Primary antioxidants, such as phenolic compounds with several hydroxyl groups (-OH), have the capacity to convert directly free radicals into stable products, by donating hydrogen or electrons [13]. On the other hand, secondary antioxidants act indirectly through different mechanisms. Some described mechanisms include the chelation of transition metals, singlet oxygen quenching, and restoration of the antioxidant activity of primary antioxidants [13]. Multifunctional antioxidants can display the properties of both primary and secondary antioxidants [13].

Therefore, a wide range of antioxidants can be added to sunscreens to perform a variety of functions. Antioxidants play an important role in the mitigation of oxidative stress in the skin, thereby reducing the signs of skin aging [14,15,16], and in the treatment of some UV-sensitive dermatosis, namely polymorphic light eruption, prurigo aestivalis, solar urticaria, and porphyria [17]. In the specific case of photodermatoses, the oral or topical administration of antioxidants aimed to neutralize the free radical species, preventing and fighting their attack on cellular structures, confirming the important role of antioxidants in UV-induced skin dermatoses [18] In fact, the skin has multiple antioxidant defense systems, including enzymatic, e.g., the glutathione-peroxidase-reductase enzyme system and superoxide dismutase (SOD) and non-enzymatic, e.g., vitamin C, vitamin E, glutathione, and coenzyme Q10 [15]. Topical antioxidants can potentially improve the intrinsic defense systems of the skin [19]. Topical delivery of antioxidants can also boost the photoprotective function of UV filters [20,21,22]. Several studies showed that some topical antioxidants used in sunscreen formulations have photoprotective properties such as reduction in erythema, sunburn cell development, and immunosuppression [21,23]. Further, several organic UV filters used in sunscreens have proved to be unstable when exposed to solar radiation, giving rise to oxidized by-products [24,25]. As a result, the phototoxicity of these compounds increases, and the photoprotective effect decreases [24,25]. Cosmetic ingredients with antioxidant activity can contribute to the stability of UV filters, reducing free radical-induced damage [22,24,25]. There is a constant search for new antioxidant compounds incorporate into cosmetic formulations, and one of the sources of election is nature [26]. Since the beginning, botanical compounds have been identified as potent antioxidants due to their polyphenolic structures [27]. With the constant innovation of the cosmetic industry, multifunctional compounds are a prerequisite in the development of new cosmetic products. More recently, the marine environment has been widely investigated as a source of natural products with interesting biological activities, including anti-aging [28], and antioxidant activities [29], resulting in the incorporation of some marine-derived products in cosmetic formulations. This work provides the reader with an up-to-date overview of the most commonly used antioxidants in commercial sunscreens and a better understanding of their mechanism of action on the photoprotective effectiveness.

## 2. Materials and Methods

### 2.1. Data Collection

The label information of a pool of 444 sunscreens, from 43 international cosmetic brands, marketed in Portuguese parapharmacies and pharmacies was collected in 2021, to assess the presence of antioxidant ingredients in sunscreens. All information available on the product labels was collected along with the information available on the manufacturers’ websites. This study was limited to cosmetics that only contained pure compounds with antioxidant activity in the skin. The analysis focused on the antioxidant ingredients with the highest usage frequency. The list of antioxidants presents in the 444 sunscreen formulations used for this study is provided in Supplementary Material (Table S1).

### 2.2. Data Analysis

The antioxidants contained in sunscreens were listed according to the International Nomenclature of Cosmetic Ingredients (INCI). The collected data were analyzed regarding the following parameters:

#### 2.2.1. Antioxidants Use

The number of sunscreen products containing pure antioxidants on their labels was evaluated and expressed as a percentage. 

#### 2.2.2. Top Antioxidants Used in Sunscreens

The antioxidants were identified from INCI lists and ranked in descending order of occurrence to disclose the top six of the most used antioxidants in sunscreens.

#### 2.2.3. Scientific Evidence Supporting the Photoprotection Effectiveness of Antioxidants

The scientific evidence for each antioxidant ingredient was searched on the online databases PubMed, PubChem, Scopus, Cochrane, and KOSMET. A broader search was performed using the keywords “INCI name” OR “synonyms”, when applicable, associated with the keywords “photoprotection”, “oxidative stress”, “antioxidant activity”, “UV-induced damage”, and “sunscreen”.

#### 2.2.4. Chemical Structures Draw

Marvin 17.21.0, Chemaxon (https://www.chemaxon.com) was used for drawing chemical structures of the top six of the most used antioxidants in sunscreens.

## 3. Results and Discussion

### 3.1. Overview of the Use of Antioxidants in Sunscreens

**Figure 1:**
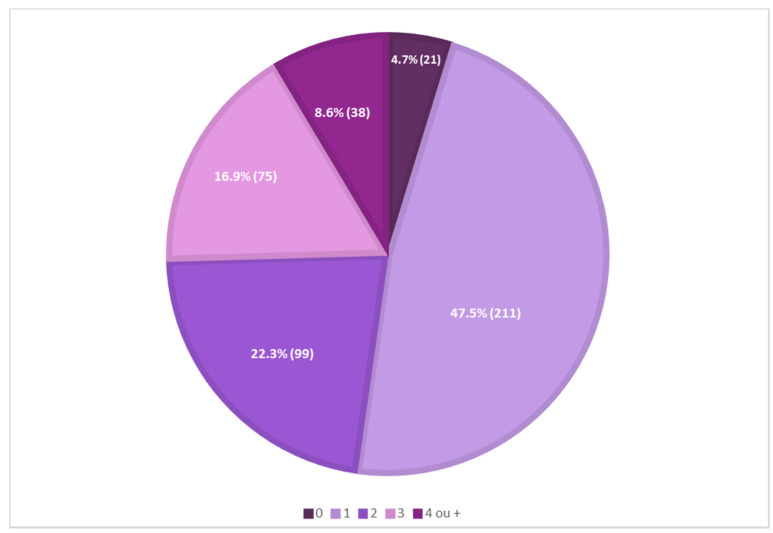
Analysis of the presence of antioxidants in 444 sunscreen formulations.

A preliminary analysis of the presence of antioxidants in 444 sunscreens commercially marketed in 2021, in a total of 43 international cosmetic brands, showed that most sunscreens (211, 47.5%) contained one antioxidant reported on their labels, and only a minor percentage of sunscreens (21, 4.7%) did not contain antioxidants reported on their labels. Mixtures of two, three, or four different antioxidants in the same formulation were also identified (Figure 1).

**Figure 2:**
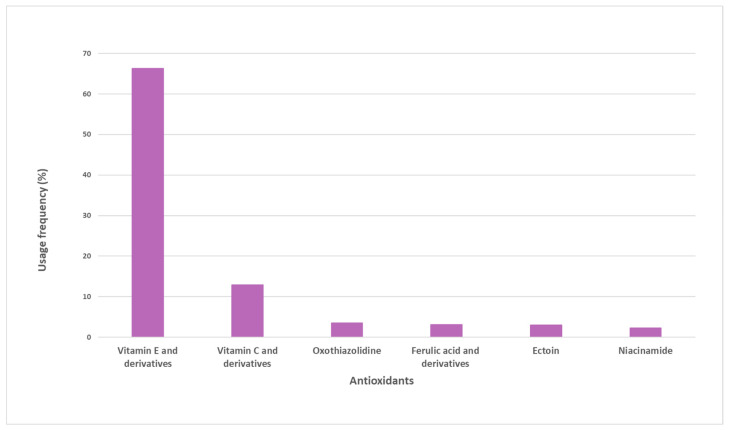
Usage frequency of the top six antioxidants with respect to the total number of occurrences on the pool of sunscreens analyzed.

A total of 38 pure antioxidants were found in the sample of analyzed sunscreens. A more comprehensive analysis was performed, and the top six pure antioxidants with the highest usage frequency (above 4%) were identified (Figure 2). Vitamin E and its derivatives (66.3%) are the most commonly used antioxidants. Vitamin C and derivatives were the second most used antioxidants, totaling a usage frequency of 12.9%. Oxothiazolidine, ferulic acid and its derivatives, ectoine, and niacinamide complete the top six of the most used antioxidants in the pool of sunscreens analyzed, whose usage frequency ranged between 4% and 7%. The usage frequency of vitamin E and its derivatives was notably higher than that of the other six antioxidants. 

**Figure 3:**
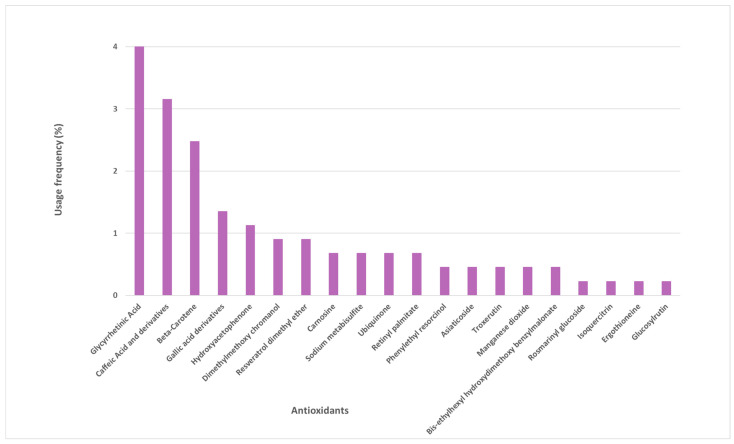
Usage frequency of the remaining antioxidants present in sunscreens.

Several other antioxidants were identified in the sunscreen labels, whose frequency of use varied between 1% and 4% (Figure 3). These are mostly naturally derived antioxidants, such as glycyrrhetinic acid, beta-carotene, caffeic acid and its derivatives, gallic acid and its derivatives, and hydroxyacetophenone. Another 15 antioxidants were found in the studied photoprotective formulations with a usage frequency inferior to 1%. 

**Figure 4:**
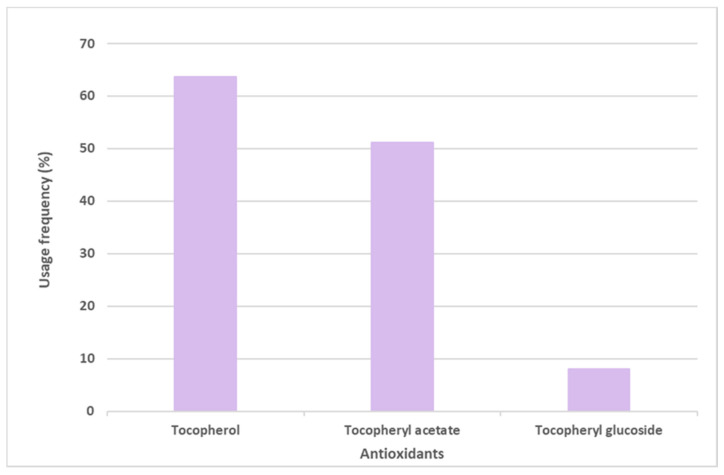
Usage frequency of vitamin E (tocopherol) and its derivatives in sunscreens.

Vitamin E and its derivative tocopheryl acetate were placed in first and second positions, respectively, in the top six most commonly used antioxidants in sunscreens. The opposite was seen with the glycosylated tocopheryl derivative (tocopheryl glucoside) which was found in a diminished number (36 formulations of the total 444 sunscreens) among all the formulations analyzed (8.1%) (Figure 4). Tocopherol (vitamin E) was used with the highest usage frequency in more than 280 sunscreens (63.7%), followed by its acetylated derivative, tocopheryl acetate (51.1%). As can be seen, more than half of the available and commercially marketed sunscreen formulations in 2021 contain tocopherol and/or tocopheryl acetate, which could demonstrate that these antioxidants were the choices preferred by the cosmetic industry for sunscreen products (Figure 4). Additionally, vitamin E and its acetylated derivative are also widely found in cosmetic products, including in a great variety of anti-aging formulations [14]. 

**Figure 5:**
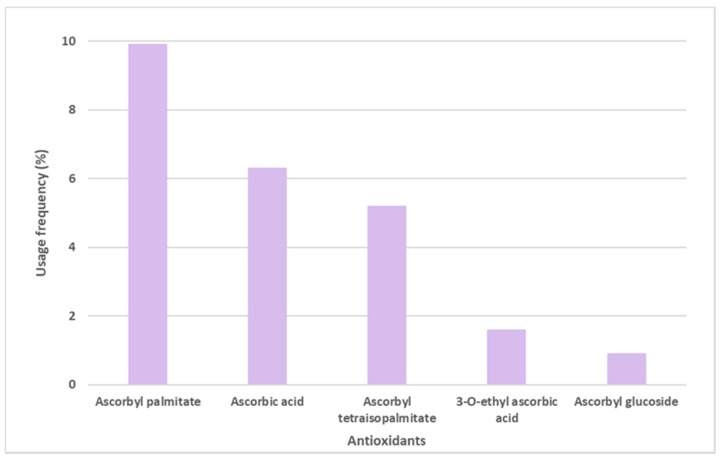
Usage frequency of vitamin C (ascorbic acid) and its derivatives in sunscreens.

Ascorbic acid, mostly known as Vitamin C, and its derivatives were also found on the labels of some sunscreens, specifically in 106 of the 444 sunscreens (Figure 5). Ascorbyl palmitate derivative, with a usage frequency of almost 10%, was the ascorbic acid derivative most frequently found on the labels of sunscreen formulations, followed by ascorbic acid (6.3%), ascorbyl tetraisopalmitate (5.2%), 3-O-ethyl ascorbic acid (1.6%), and finally the glycosylated derivative of ascorbic acid, ascorbyl glucoside, with a usage frequency <1%. Compared to vitamin E and its derivatives, the usage frequency of vitamin C and its derivatives is five times inferior to the previous one. This could be explained by the fact that, in contrast to vitamin E, a fat-soluble molecule, vitamin C is a hydrophilic compound, and is thus challenging to introduce into cosmetic formulations. 

**Figure 6:**
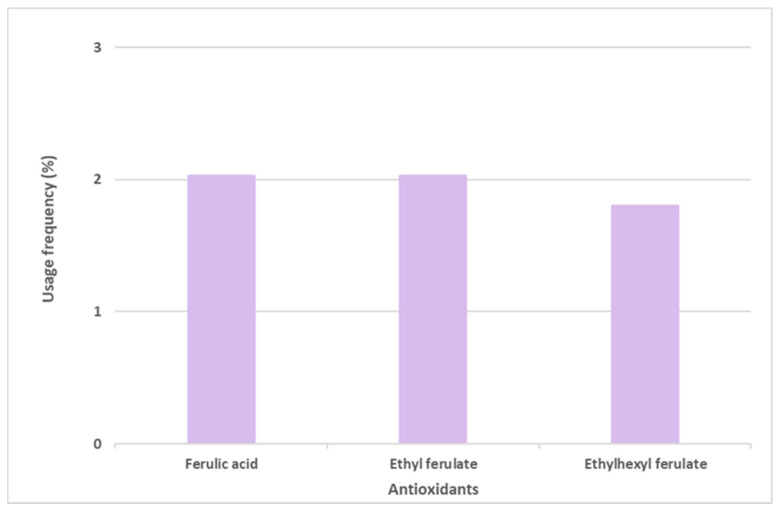
Usage frequency of ferulic acid and its derivatives in sunscreens.

Ferulic acid and its derivatives, ethyl ferulate and ethylhexyl ferulate, are also among the top six most commonly used antioxidants in sunscreen formulations (Figure 6). Ferulic acid and ethyl ferulate are both present in nine of the 444 photoprotective formulations studied, with a usage frequency of 2.0%, and ethylhexyl ferulate was only found in eight of the 444 total analyzed sunscreens (1.8%). These hydrophobic derivatives demonstrate more stability in oxidative processes when compared with the parent compound, ferulic acid [30]. However, a similar usage frequency of all three compounds belonging to the ferulic acid derivatives category was noticed. Considering that these compounds are cinnamates, they tend to undergo photoisomerization in the presence of UV radiation [31]. Overall, ferulic acid and derivatives (5.9%) were not as widely used in photoprotective formulations as vitamin E and derivatives (66.3%) and vitamin C and derivatives (12.9%).

Interestingly, Silva and Ferreira et al. analyzed and characterized the antioxidant compounds present in anti-aging formulations over a seven-year period, and vitamin E and its derivatives were the most used [14]. Tocopherol derivatives were more frequently found than tocopherol itself [14]. Herein, vitamin E and its derivatives were also the most used antioxidants in sunscreen products; however, tocopherol (63.7%) was more frequently used than tocopheryl acetate (51.1%) in sunscreens. Ascorbic acid and ascorbic acid derivatives were widely used in anti-aging formulations, with usage frequencies of 11.0% and 20.0%, respectively [14]. In sunscreens, both were used in lower percentages, probably because they have other biological activities, such as the promotion of collagen synthesis, and are more relevant to anti-aging products [14]. Niacinamide was the antioxidant with the fourth highest usage frequency (5.3%) in the top ten of the most used antioxidants in anti-aging products [14]. Herein, niacinamide was only found in 4.3% of the total sunscreens studied, reaching the last place in the top six of the most used antioxidants in sunscreens. It is possible to denote the presence of antioxidants in both categories of cosmetic products, anti-aging and sunscreen formulations, with vitamin E and vitamin C and their derivatives and niacinamide being the most used antioxidants in both cosmetic formulations, which could suggest the multifunctional action of these antioxidants. Oxothiazolidine and ectoine were detected only on sunscreen labels, and ferulic acid and its derivatives were found in anti-aging cosmetic formulations with a usage frequency lower than 1% [14], compared with their usage frequency of almost 6% in sunscreen products recently marketed.

### 3.2. Scientific Evidence Supporting the Photoprotection Effectiveness of the Top Six Antioxidants 

#### 3.2.1. Vitamin E and Derivatives

**Figure 7:**
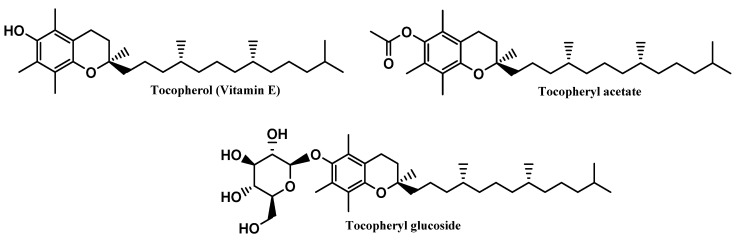
Chemical structure of vitamin E and its derivatives.

Tocopherol, a naturally occurring lipophilic vitamin, is widely found in fruits, vegetables, and seeds, and has been reported for its strong antioxidant activity [32], as a scavenger of ROS, namely peroxyl radicals, preventing the oxidation of biomolecules such as proteins and lipids [32], as well as its cytoprotective activity. Additionally, tocopherol was also reported for inhibiting the activity of protein kinase C (PKC) and its mediated pathways, suggesting its beneficial effects in several pathologies, including diabetes mellitus and cardiovascular and inflammatory diseases [33,34,35,36]. Tocopherol reveals poor chemical and photo-stability, along with a particular susceptibility to oxidation by alkoxyl radicals, resulting in the subsequent formation of chromanoxyl radicals [15]. For that reason, the development of new derivatives obtained through molecular modifications on the original compound are urged, namely tocopherol acetate and tocopheryl glucoside [15] (Figure 7). While tocopherol acetate results from the acetylation of the free aromatic hydroxyl group of α-tocopherol, tocopheryl glucoside results from the addition of a glucose sugar unit [14,37]. These pro-vitamins do not have any activity, by themselves, requiring bioactivation by cutaneous phosphatases or esterases to release in situ the free active vitamin E [38,39,40]. Moreover, due to its high hydrophobicity, tocopherol has poor topical formulation and aqueous solubility, which could potentially be overcome by the use of both pro-vitamins [41]. Even though tocopheryl acetate presents a more lipophilic character than its parent compound, the higher stabilization, and less probability of being oxidized, along with its insertion in the skin phospholipid bilayer, potentiate the neutralization of free radicals [15,42,43]. On the other hand, tocopheryl glucoside turns into a more lipophilic and active molecule–free tocopherol–after cleavage of the glycosidic bond, catalyzed by β-glucocerebrosidase in the stratum corneum [37].

There are several studies supporting the multifunctional antioxidant and photoprotection efficacy of vitamin E. A single-center, open, placebo-controlled intra-individual study carried out on 30 patients showed that pre-treatment of photosensitive sites with a topical vitamin E formulation significantly reduces photosensitivity [44]. In addition, the results achieved with all irradiation-induced reactions in patients treated with vitamin E or a simple vehicle indicated that formulations containing vitamin E are a promising approach to prevent photoinduced skin damage [44]. Many in vivo studies performed in albino mice revealed that α-tocopherol provided significant protection against skin oxidative stress induced by UVB radiation, possibly due to an upregulation of a network of enzymatic and non-enzymatic antioxidants [45,46]. Moreover, the photoprotection provided by tocopherol seems to result from its ability to partially absorb UVB radiation [47]. In human keratinocytes exposed to UVA radiation, the application of tocopherol also provided photoprotection by increasing glutathione production and reducing lipid peroxidation and ROS and malondialdehyde (MDA) levels [42,43]. Tocopheryl acetate is the second most prevalent antioxidant used in sunscreen formulations. Some authors argue that there is no significative esterase activity to convert tocopheryl acetate into its active form [48,49]. In fact, according to a study performed on human volunteers, the epidermal and dermal layers of human skin absorb tocopherol acetate less than α-tocopherol, and there is little conversion of tocopherol acetate to free α-tocopherol [50]. Nevertheless, some in vivo studies conducted in albino mice revealed an increase in tocopherol skin levels, emphasizing the protection against skin damage, albeit less significant than that provided by tocopherol application, possibly as a result of its reduced UVB absorption [47,51]. Further studies are required to clarify the photoprotective effectiveness of tocopheryl acetate, as well as to improve its delivery and bioactivation in human skin. Although tocopheryl glucoside is the vitamin E derivative less used in sunscreen formulations, some studies were performed to confirm its multifunctional antioxidant and photoprotective effectiveness. A study developed in both reconstituted human epidermis and viable human skin has shown that tocopheryl glucoside reveals a higher percentage of metabolization to the active form (α-tocopherol) than tocopheryl acetate, even though skin diffusion was slower [37]. In fact, tocopheryl glucoside has been demonstrated to produce a significant reservoir effect, associated with a progressive supply of free tocopherol, first in the stratum corneum and then in the other skin compartments, conferring protection for at least 24 h [37,38].

Vitamin E acts as a direct antioxidant against singlet oxygen and superoxide anions. Additionally, when lipid peroxidation occurs in cell membranes, tocopherol’s function as a “chain breaker” is reported, thus preventing lipid peroxidation by scavenging peroxyl radicals [52,53]. The ongoing renewal of vitamin E by other biological agents is essential to preserve its antioxidant capabilities. For that purpose, ascorbic acid and glutathione are both necessary for the prolonged activity of vitamin E as they provide the requisite hydrogen ions when the tocopherol radical is produced [54]. Tocopherol also enhances collagen synthesis while preventing collagen degradation by lowering MMP levels and maintaining tissue inhibitors of MMP expression, thus preserving the dermis’ integrity [55,56]. Moreover, acute and chronic UV-mediated skin reactions such as erythema and edema, sunburn-cell formation, DNA photo-adduct creation, immunosuppression, and photocarcinogenesis have been shown to be effectively reduced by topical application of vitamin E [57,58].

#### 3.2.2. Vitamin C and Derivatives

**Figure 8:**
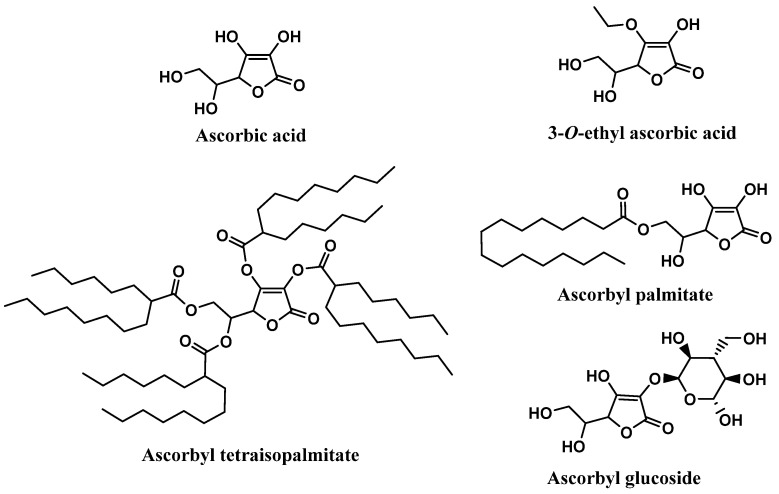
Chemical structure of vitamin C and its derivatives.

Ascorbic acid, chemically known as (*5R*)-[(*1S*)-1,2-dihydroxyethyl]-3,4-dihydroxyfuran-2(5H)-one, extensively recognized as vitamin C, is widely found in fresh fruits and vegetables [59,60]. Vitamin C has been reported to have health-promoting effects and benefits in several pathologies, such as cancer, diabetes, chronic inflammation, and cardiovascular, neurological, and skin diseases [61]. Among all the biological activities, antioxidant activity is highly frequently reported, due to its effective neutralization of ROS and interruption of lipid peroxidation chain reactions [14,62]. Vitamin C is also capable of regenerating oxidized vitamin E [63,64]. Therefore, ascorbic acid, the most biologically active form of vitamin C, has the ability to reduce several free radicals such as superoxide, hydroxyl, alkoxyl, peroxyl, as well as tocopheroxyl radicals [59,65,66]. Although it is used as an active ingredient in diverse cosmetic products, it is chemically unstable, which raises some obstacles in the development of cosmetic formulations, especially aqueous formulations [63,67]. To overcome this limitation, the synthesis of ascorbic acid derivatives with desirable characteristics has been explored. The majority of these derivatives were obtained through the esterification of one or more hydroxyl groups present in the vitamin C structure with small (3-O-ethyl ascorbic acid), or long organic chains (ascorbyl palmitate and ascorbyl tetraisopalmitate) (Figure 8). In fact, ascorbyl tetraisopalmitate possesses all the hydroxyl groups esterified with the long organic chain of palmitic acid, and together with ascorbyl palmitate, substituted in only one hydroxyl group, constitute the vitamin C derivatives that have higher lipophilic character than the parent compound [14]. Those derivatives could also promote easy skin absorption [68]. Ascorbyl glucoside is the glycosylated derivative of vitamin C, which is more suitable for aqueous formulations, and it seems to have a lower skin penetration, releasing ascorbic acid in the skin after 24 hours [68]. All the ascorbic acid derivatives are regarded as more stable than the parent compound, and like the vitamin E derivatives, vitamin C derivatives could be hydrolyzed by skin enzymes, releasing pure vitamin C in the skin [69]. 

Ascorbic acid was already reported for its photoprotective effects against UVA and UVB irradiation on human skin fibroblasts [70], keratinocytes [71], and stratum corneum lipids [72]. Ascorbic acid also showed a significant increase in the skin deposition of mineral UV filters without enhancing their skin permeation, increasing the durability of sunscreen on the skin and consequently improving sun protection [73]. An in vivo study using porcine skin demonstrated that vitamin C protects the skin from UV-induced damage, reducing erythema and sunburn cell formation [74]. Topical ascorbic acid was shown to significantly reduce the incidence of skin tumors in hairless mice after chronic exposure to UVR [45]. In a double-blind randomized trial performed for 6 months, where the action of the 5% of vitamin C cream and control (excipient) on photoaged skin was compared, ascorbic acid led to a clinical improvement of the photoaging skin signs [75]. Ascorbyl palmitate shielded the porcine skin from UV-induced free radicals [76]. However, two different studies found that ascorbyl palmitate did not protect mouse skin from UVB-induced photoaging [45], and may also promote UVB-induced lipid peroxidation and cytotoxicity in human keratinocytes, which consequently exacerbates skin damage [62]. This is a result of the oxidation of the lipid moiety of ascorbyl palmitate which can form UVB-induced free radical metabolites [62]. So, more studies are needed to clarify these results. Ascorbyl tetraisopalmitate also boosts cell tolerance to UVB exposure [77], prevents UVA-induced damage in human keratinocytes and melanoma cells [78], enhances collagen production and intracellular concentration of ascorbic acid, as well as inhibiting MMP activity [78]. An ex vivo study demonstrated that the topical application of ascorbyl tetraisopalmitate in human skin explants decreased the sunburn cells formulations, increased the procollagen type I, and upregulated the expression of tropoelastin expression when compared with the vehicle [79]. Ascorbyl glucoside also limits UV-induced damage of human skin keratinocytes [80,81], fibroblasts [80], and of a human reconstructed epidermal model [68]. Compared to ascorbic acid, 3-O-ethyl ascorbic acid had significant and prolonged DPPH free radical scavenging action (0.032 g/L) [82,83,84]. Limited data are available concerning in vitro and in vivo studies with 3-O-ethyl ascorbic acid, thus more research is required to assess its effectiveness as a photoprotection booster [68,85]. 

Ascorbic acid can function as a free radical scavenger, an antioxidant that neutralizes ROS, and a reducing agent for enzymatic processes. For these purposes, ascorbic acid donates a single reducing equivalent, forming the radical monodehydroascorbate, which reacts preferentially with other radicals generated during the oxidative stress process, being oxidized to dehydroascorbate [59]. Thus, vitamin C can inhibit elastin synthesis and the activation protein-1 (AP-1), leading to a reduction in MMP production and collagen damage [21,86]. This vitamin also prevents the reduction in CD1a-expressing Langerhans cells after UV exposure [87]. The mechanism of vitamin C photoprotection also includes the reduction in erythema, sunburn cell formation, and immunosuppression [21].

#### 3.2.3. Oxothiazolidine

**Figure 9:**
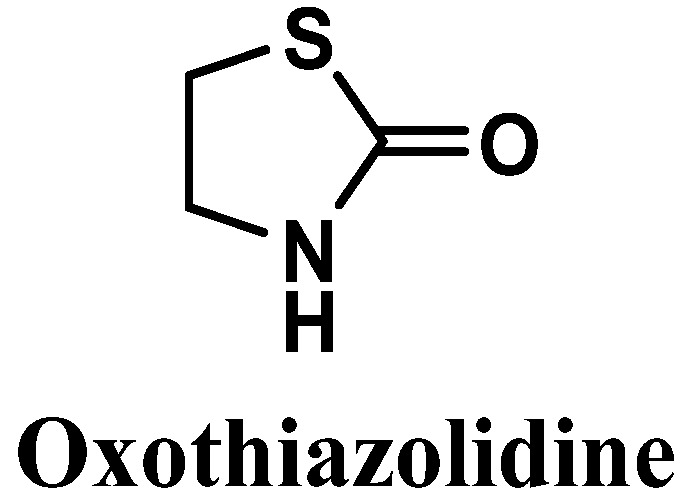
Chemical structure of oxothiazolidine.

Oxothiazolidine (1,3-thiazolidin-2-one) is a heterocyclic compound with interesting antioxidant activity [88] (Figure 9). It has been reported to possess good skin and cell penetration and the ability to protect the skin against IR- and UV-induced alterations, control accelerated skin aging, and preserve the epidermis, dermis, and dermal-epidermal junction (DEJ) [88,89,90]. 

Some studies have confirmed the multifactorial antioxidant and photoprotective activities of oxothiazolidine. The antioxidant activity has been reported as noteworthy considering its actions regarding a large panel of ROS, including superoxide anion, hydrogen peroxide, and hydroxyl radicals [91]. In 2008, Lafitte et al. evaluated oxothiazolidine regarding its ability to release taurine locally in the skin under oxidative stress conditions [90]. Taurine is a naturally occurring amino acid with a confirmed preventive effect against UVB-induced skin damage [90]. The novel ingredient has shown antioxidant and electrophilic scavenging properties [90]. Preliminary studies evidenced the photoprotective effect of oxothiazolidine against UVA-induced oxidative stress, and its activity as a scavenger of electrophilic species, such as toxic aldehydes, usually formed during lipid peroxidation reactions with lipids present in the cellular membrane [90]. Taurine is a compound naturally present in human skin, and in situ taurine production was already reported to play an important role in photoprotective responses by limiting the UVR-induced cellular apoptosis [92] and immunosuppression process [93]. Oxothiazolidine was also reported to display photoprotective potential, preservation of collagen VII in DEJ, reduction in MMP-1, cyclooxygenase-2 (COX-2), and decorin expression. Furthermore, its ability to reach the epidermis and dermis skin layers was demonstrated, using in vitro three-dimensional model and ex vivo human skin explants [89]. Recently, Jacques et al. published a study where the scientific proof of the sustained effect of oxothiazolidine as an antioxidant to be used in sun protection cosmetic products was made [38]. A formulation containing the antioxidant oxothiazolidine was prepared and investigated for its dermal bioavailability and antioxidant properties [38]. An immediate complementary sunlight protective action of oxothiazolidine was attained after topical application, due to its fast penetration and fast reaction with ROS to give taurine, which was detected 30 minutes post-UV irradiation [38]. Indeed, oxothiazolidine and taurine possess antioxidant, protective, and anti-aging properties, with oxothiazolidine being more potent than the final compound taurine, regarding its antioxidant potential [38]. Other derivatives of oxothiazolidine have been investigated, and there are some registered patents of those derivatives for skincare [94], promotion of desquamation of the skin [95], topical use against oxidative stress consequences [91], and as active protective agents [96].

From a mechanistic point of view, oxothiazolidine reacts with ROS and undergoes successive oxidation reactions, culminating in the opening of the cyclic portion of oxothiazolidine to give the taurine-free form [90]. None of the oxidized intermediates were detected by high-performance liquid chromatography (HPLC) analysis, suggesting that the intermediates are unstable; thus, the oxidation of oxothiazolidine only stops when the taurine compound is achieved [90], which corroborates the hypothesis of the mechanism of action of oxothiazolidine. Oxothiazolidine acts in the neutralization of toxic and reactive aldehydes presented as end products of lipid peroxidation [90]. It is achieved through oxothiazolidine reaction with the cytotoxic aldehyde functional groups of those compounds, given a more stable adduct, carbinolamine [90].

#### 3.2.4. Ferulic Acid and Derivatives

**Figure 10:**
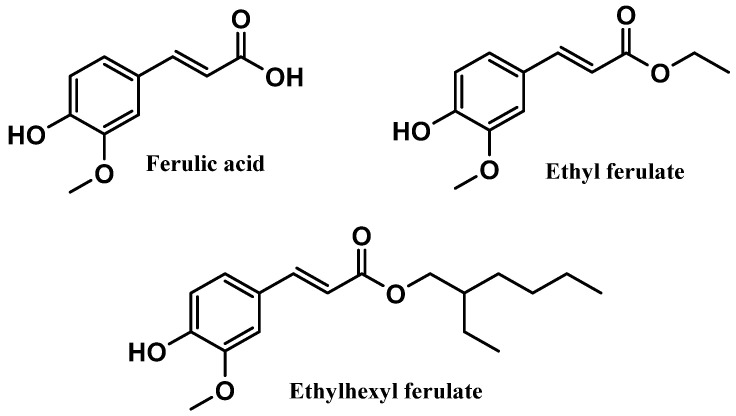
Chemical structure of ferulic acid and its derivatives.

Ferulic acid, chemically known as 4-hydroxy-3-methoxycinnamic acid, is a naturally occurring phenolic acid present in several plants, including rice, oats, pineapple, grains, flowers, fruits, coffee, peanuts, and nuts [97]. These natural products can be found in their free form or conjugated with sugar, lipid, and protein structures [97]. Natural ferulic acid has been described for its anti-inflammatory [98], antimicrobial [99], antitumor [100], neuroprotectant [101], and antioxidant activities [102,103], as well as for uses in cosmetic products [97,104,105]. The effective action of ferulic acid as a free radical scavenger has been widely reported, specifically for superoxide anion radical and peroxyl radical, avoiding oxidative stress processes, and inhibiting lipid peroxidation, respectively [102,106]. This antioxidant response could be attributable to the aromatic hydroxyl group present in the structure of the naturally derived phenolic acid [102,106]. Ethyl ferulate and ethylhexyl ferulate are two ester derivatives obtained by the introduction of organic chains to the parent compound, ferulic acid, presenting a more hydrophobic character (Figure 10). Ferulic acid was already reported for its in vitro lipid peroxidation inhibitory effect, as well as the scavenging activity of superoxide and hydroxyl radicals, terminating radical chain reactions [103,107]. Like ferulic acid, ethyl ferulate and ethylhexyl ferulate were already reported for their anti-inflammatory and antioxidant activities and ability to absorb UVR [108,109].

In vivo and in vitro studies were performed to access the photoprotective action of phenolic acids, including ferulic acid [104]. Adult human skin samples were used to evaluate the protective effect of ferulic acid against UVB radiation, which was also demonstrated to reduce UVB-induced erythema [104]. Skin absorption of this compound was not influenced by the variation of the pH, thus presenting a similar skin penetration rate in acidic and neutral pH [104]. The in vivo SPF value of a sunscreen formulation containing two UV filters, ethylhexyl triazine (5.00%) and bis-ethylhexylmethoxyphenol methoxyphenyltriazine (10.0%), and ferulic acid (1.00%) was determined and compared to a similar formulation without ferulic acid [110]. Ferulic acid was demonstrated to enhance the in vivo SPF value of the formulation (from 19.7 to 26) and to have protective effects against UV-induced erythema [110]. A similar study confirmed the potential of ferulic acid to boost the photoprotective response of a sunscreen containing the same UV filters, and also increased the UVA protection factor by 26% [111]. Interestingly the photoprotective role of this naturally derived cinnamic acid was already discussed, where its UV filtering and antioxidant activity was confirmed, namely in ROS scavenging action, thus protecting skin from visible light and UV-induced oxidative stress events [112]. Ferulic acid is also characterized by high absorption of UVR and radiation-initiated antioxidant potential [103], inhibitory action towards the UVA-induced melanogenesis in the skin, and the protective effect of Nrf2 towards UVA-mediated oxidative stress [113]. Indeed, a patent was registered, mentioning ferulic acid ester derivatives as useful for cosmetic applications due to their proven antioxidant and UV absorbent activities [114]. 

UV-catalyzed radical-scavenging characterizes the mechanism of action of ferulic acid [103]. By the abstraction of the electrons of radicals, a phenoxyl radical is formed; however, owing to its lack of reactivity, the phenoxyl radical cannot initiate or contribute to the propagation of radical chain reactions [103]. Ferulic acid can also inhibit melanin formation, acting as a competitive inhibitor of tyrosinase enzyme [104,113], and protecting against UVB-induced erythema [97,104].

#### 3.2.5. Ectoine

**Figure 11:**
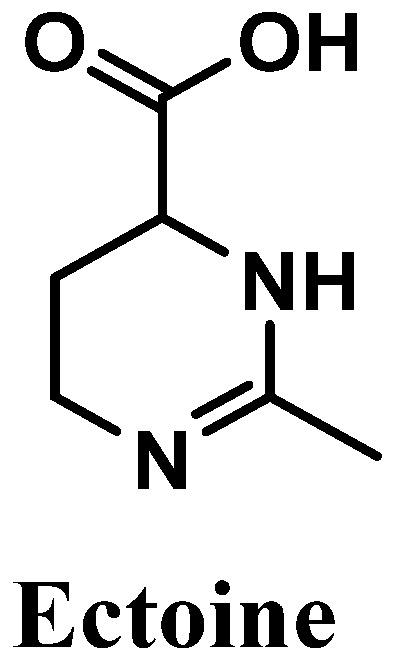
Chemical structure of ectoine.

Ectoine, chemically known as (*6S*)-2-methyl-1,4,5,6-tetrahydropyrimidine-6-carboxylic acid, is an amino acid derivative isolated from marine bacteria and algae that live under extreme conditions [115,116,117] (Figure 11). Reported for its antioxidant potential, ectoine possesses high ROS scavenger activity, especially towards hydroxyl radicals [118]. This amino acid derivative is biosynthesized in order to protect organisms’ organelles and biomolecules against dehydration, which is caused by drastic variations in salt concentrations/deficit of water, affecting the osmotic equilibrium, and high temperatures [119]. Skin is a physical barrier that is constantly exposed to several external aggressors, with frequent variations in temperature and excessive UVR exposure [120]. Therefore, ectoine could be used for diverse cosmetic products, including sunscreens and products for dry skin [115,121], acting as a good protective agent. 

The in vivo investigation of the beneficial properties of ectoine is underexplored compared with several in vitro studies performed to evaluate the antioxidant and photoprotective potential of this compound. One clinical study about the anti-aging and UV-protective effects of ectoine and its application for the development of skin care products was found [122]. A total of 104 healthy female volunteers participated in a randomized and double-blind application test study, aiming to evaluate the compatibility and the efficacy of ectoine (using a cosmetic formulation containing 2% of ectoine) in comparison to a vehicle emulsion, which corroborates the protective effects and UV-induced anti-aging properties of ectoine [122]. Additionally, a moisturizing effect of ectoine was also confirmed [122]. It was also shown that the topical application of ectoine decreased the negative effects of excessive exposition to UVR [123]. The ability of ectoine to react with hydroxyl free radicals was also reported [116]. Two major products, N-acetamide aspartate and N-acetimide-β-alanine, were produced by the reaction between ectoine and hydroxyl radicals, showing the scavenger potential of this compound in neutralizing species involved in oxidative stress processes [116]. The decrease in Langerhans cells, which could be induced by solar radiation, was reduced by ectoine application before sun exposure [123]. Ectoine was also confirmed as an effective natural compound that could be used in the prevention of premature aging induced by solar radiation, specifically UVA radiation, through the suppression of AP-2, the decrease in overexpression of intercellular adhesion molecule 1 (ICAM-1) in keratinocytes, and the inhibition of the formation of photo-induced mitochondrial DNA mutations in dermal fibroblasts [115]. The photoprotective potential of sunscreens containing naturally derived compounds was tested, and ectoine demonstrated a maximal protective effect of 92.7% and 68.9% against visible light and UVA/visible light, respectively, at a maximum concentration of 0.1 mM [124]. A similar study demonstrated the photoprotective effects of ectoine in UVA-induced oxidative damage in dermal fibroblasts [125]. Indeed, it was also proved that this molecule upregulated the expression of diverse genes associated with AKT/PI3K signaling pathways, decreased ROS levels, and increased the levels of enzymatic and non-enzymatic antioxidants, superoxide dismutase, and glutathione, respectively [121,125]. In addition, ectoine prevented the formation of ceramides due to its quenching proprieties for singlet oxygen and, consequently, avoided the initiation of inflammatory processes [103,115].

The mechanism underlying the protective effect of ectoine against UVR has recently been studied. It is known that ectoine reduces the expression of proinflammatory agents, restraining the initiation of inflammatory processes [115,126]. Further, it was hypothesized and confirmed that the production of heat shock proteins is activated, which leads to a protective response against UVR [124,126]. Ectoine also revealed quenching properties of UV-induced ROS, namely singlet oxygen species [115,119]. More studies are required in order to better understand the mechanism of action of ectoine, as a photoprotective agent that has been used in sunscreens formulations.

#### 3.2.6. Niacinamide

**Figure 12:**
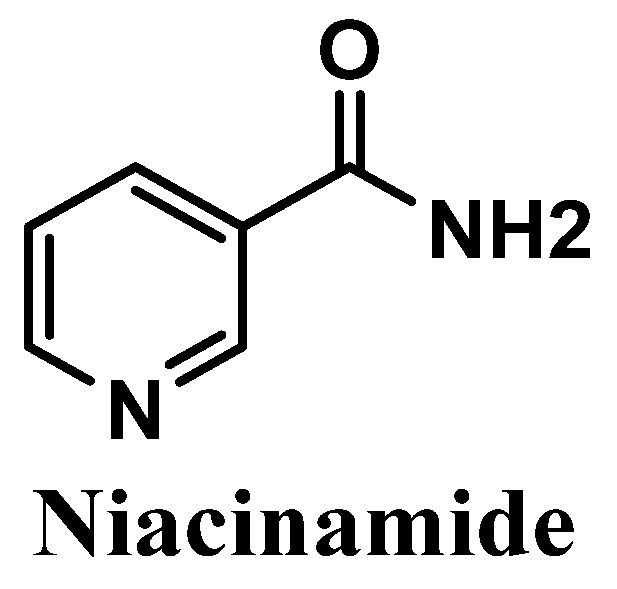
Chemical structure of niacinamide.

Niacinamide, also called nicotinamide, commonly known as the active form of vitamin B3, is an amide of the carboxylic acid of water-soluble niacin, and an essential component of the coenzyme nicotinamide adenine dinucleotide (NAD) [127,128] (Figure 12). It is easily obtained from natural sources such as potatoes, bananas, grains, turkey, and tuna [129]. Anti-inflammatory and antioxidant activities of niacinamide have already been reported, showing its efficacy in the treatment of several skin pathologies, namely rosacea, acne [130,131], and dermatitis [130,132,133]. Niacinamide also reduces transepidermal water loss (TEWL) [134] and suppresses the transference of the melanosomes to keratinocytes, which consequently reduces hyperpigmentation [130,135]. 

Three distinct clinical studies were already performed to support the protective effects of niacinamide against the UVR negative effects on the skin. Several clinical studies have been conducted to confirm the efficacy of the use of topical niacinamide in the treatment of photoaging in Asian [136,137] and Caucasian [138] groups of women. These studies demonstrated the protective effect of niacinamide against UV-induced immunosuppression and the suppression of UV-induced melanosome transfer [136], contributing to its anti-photoaging action and mitigating the hyperpigmentation effects, respectively [135,137]. The significant decrease in pro-inflammatory interleukins, after the cells treatment with niacinamide [130], and the activation of DNA repair mechanisms in cases of UV-induced skin damage [139] were also confirmed by in vitro studies. The amide derivative of niacin was already reported for its protective effects against UV-induced skin aging and oxidative stress in epidermal keratinocytes [139,140,141], in human dermal fibroblasts [142], and in melanocytes [139,143] cell lines. Oral ingestion of niacinamide helps to avoid UV-mediated immunosuppression and helps to maintain the levels of NAD controlled, thus avoiding the loss of efficiency of the DNA repair mechanisms [129]. Accordingly to Sivapirabu et al., niacinamide could be used as an interesting agent in the optimization of the photoprotection of sunscreens, acting in the prevention of the immunosuppression effects induced by both UVA and UVB radiation [144]. This study investigated the effects and the mechanisms underlying the topical application of niacinamide, and the results achieved proved that niacinamide increased the pool of enzymes involved in cellular metabolism, crucial for the protection against UV-mediated immunosuppression [144]. Niacinamide was also confirmed as an anti-pollution agent, in which keratinocytes were protected from particulate matter 2.5 (PM2.5)-induced oxidative stress in the skin [145]. Recently, two products were studied, which combined niacinamide with ZnO nanoparticle composite [146], and a niacinamide/jojoba oil hybrid nanogel [147], both with photoprotective effects and application as cosmetic products. The niacinamide coating of the ZnO nanoparticle composite demonstrated lighting effects and diminished the transmission and high reflectance against UVR, ensuring adequate UV protection [146]. Thus, the coating of niacinamide could be effective for the development of new sunscreen formulations [146]. The nanogels demonstrated protective action towards the human epidermal keratinocytes against UVR, and consequently can be added to sunscreen products in order to improve skin photoprotection [147].

The mechanism of action of niacinamide involves the prevention of immunosuppression induced by both UVA and UVB radiation and by increasing the enzymatic and non-enzymatic antioxidants in the skin [144], which can be used to optimize the photoprotective response of sunscreens. The reduction of immunosuppression, anti-photoaging action [144], and activation of DNA repair mechanisms [139] could supplement the photoprotective action of niacinamide.

## 4. Summary of the Mechanisms of Action of the Antioxidant in Sunscreens

The antioxidants investigated protect skin from a wide spectrum of UVR-related deleterious effects, being the main outcome of the in vivo studies the decrease in UV-induced erythema. Regarding the mechanism of action, several research studies corroborate their antioxidant action, as radical scavengers of UV-induced ROS and RNS (all the antioxidants), including hydroxyl radical (oxothiazolidine, ectoine, vitamin C, and ferulic acid and their derivatives), superoxide anion (oxothiazolidine, vitamin C, and ferulic acid and their derivatives), hydrogen peroxide (oxothiazolidine, and vitamin C), peroxyl (vitamins E and C, ferulic acid and their derivatives), peroxynitrite anion (vitamin C), singlet oxygen (ectoine, and vitamin C), and toxic intermediates, namely reactive aldehydes (oxothiazolidine). Additionally, some of them increase the levels of antioxidants associated with enzymatic and non-enzymatic systems, such as vitamin E and derivatives, ectoine, and niacinamide. Other mechanisms of action noted were the activation of DNA repair mechanisms in epidermal and dermal skin cells (oxothiazolidine, ectoine, and niacinamide), inhibition of the immunosuppression processes (vitamins E and C, oxothiazolidine, and niacinamide), decrease in Langerhans cells (vitamin C, and ectoine), reduction in the release of pro-inflammatory interleukins (ferulic acid, and ectoine), and metalloproteinases (vitamins E and C, and oxothiazolidine), tyrosinase inhibition (ferulic acid, and niacinamide), as well as a decrease in the effects of sunburned cells (vitamins E and C). It is noteworthy that all antioxidants possess a multifunctional mechanism of action. However, the absence of comparative studies regarding the potency of the compounds restrains the conclusion about the most potent and effective compound.

## 5. Conclusions

The adoption of protective measures is essential to avoid sunburn, photoaging, and skin diseases induced by excessive solar exposure, which promotes the generation of ROS and RNS, thus inducing oxidative stress. Sunscreens incorporate UV filters, frequently combined with antioxidant compounds. In addition to stabilizing formulations and UV filters, antioxidants can be used to boost the skin photoprotection effect. An up-to-date overview of the most commonly used antioxidant compounds, in a total of 444 commercial sunscreens available in the Portuguese market, as well as a compilation of the scientific evidence regarding the photoprotective effectiveness of the most widely used antioxidants, was herein performed. More than 95% of the sunscreen formulations contained at least one antioxidant compound. This widespread use may be justified by the well-known key role of UV radiation in generating skin oxidative stress. The majority of the antioxidants are naturally derived, mostly from terrestrial sources, with the exception of ectoine, a marine-derived compound, and oxothiazolidine, obtained by synthesis. Vitamin E and its derivatives were the most used antioxidants in sunscreens, with usage frequency above 65%, followed by vitamin C and derivatives (12.9%). One antioxidant among the top six (ectoine, 5.6%) is derived from marine organisms, bacteria, and algae, showing the potential of marine-derived compounds as an interesting source for the discovery of compounds to be incorporated in sunscreens. Oxothiazolidine (6.5%), ferulic acid and its derivatives (5.9%), and niacinamide (4.3%) completed the list. By comparing these with the antioxidants commonly present in anti-aging formulations, some conclusions can be drawn. Only 77 % of anti-aging products included antioxidants. Vitamins E and C and their derivatives were present in both cosmetic formulations with the highest frequency of use. Ferulic acid’s usage frequency was six times higher in sunscreen products than in anti-aging formulations. Ectoine and oxothiazolidine were only found in sunscreens. Vitamin C and niacinamide were mostly found in anti-aging cosmetics, probably because of their well-known role in the promotion of collagen synthesis and depigmenting action, respectively. 

The photoprotective action and antioxidant activity of the molecules were also compiled based on scientific evidence reported in the literature. The existence of a plethora of scientific works confirming the effectiveness and safety of these antioxidants is possibly the main factor that contributes to the vast use of those antioxidants in cosmetic products, including sunscreens. For all the antioxidants used in sunscreens belonging to the top six, at least one in vivo and/or clinical study was found. Vitamins E and C, ferulic acid and their derivatives, as well as niacinamide, were the four antioxidants with the most in vivo and/or clinical studies reported. In some cases, namely for vitamin E and vitamin C derivatives, some in vitro contradictory results were found regarding their antioxidant and photoprotective effectiveness. Oxothiazolidine and ectoine were the antioxidants with fewer in vivo and/or clinical studies, showing the importance of more future research with these two pure antioxidants for a better understanding of their applicability in photoprotective formulations. Together with ferulic acid, these three compounds were only included in 2010 in the inventory of cosmetic ingredients in Cosing, a European Commission cosmetic ingredients database, with the last update in May of 2019 (ferulic acid) and November of 2022 (oxothiazolidine and ectoine). This might help explain the lower number of studies described in the literature.

To conclude, this work identified the antioxidants present in commercial sunscreens and their photoprotective action, unveiling the main mechanisms of action underlying their effectiveness. Moreover, with this insight, the cosmetic industry could invest in the most promising antioxidant compounds, designing new sunscreen formulas to boost the effectiveness of UV filters. 

## 6. Strengths and Limitations

This study was performed for the Portuguese market, although the studied products were mostly from international brands, and included only sunscreens marketed in pharmacies and parapharmacies. This work contains detailed information on the composition of sunscreens, which is a result of an exhaustive and comprehensive analysis of the label information, and research of the scientific evidence in diverse databases, contributing to the robustness of the insight presented. The information collected was organized and subjected to a critical appraisal, unveiling an original and up-to-date overview of the use of antioxidants in sunscreens. Some disparities can be found when compared to studies performed in other markets, especially non-EU countries, or other distribution channels. Another point that could contribute to the limitations of this work is the in-house studies of cosmetic industries, which are not available in the usual databases and/or in online sources, thus limiting sight of the full picture.

## Data Availability

Not applicable.

## Supplementary Materials

The following supporting information can be downloaded at: https://www.mdpi.com/article/10.3390/antiox12010138/s1, Table S1: CVS-document containing the list of the antioxidants present in the 444 sunscreens formulations.
